# Genetic contributions to brain serotonin transporter levels in healthy adults

**DOI:** 10.1038/s41598-023-43690-x

**Published:** 2023-09-30

**Authors:** Silvia Elisabetta Portis Bruzzone, Arafat Nasser, Sagar Sanjay Aripaka, Marie Spies, Brice Ozenne, Peter Steen Jensen, Gitte Moos Knudsen, Vibe Gedsoe Frokjaer, Patrick MacDonald Fisher

**Affiliations:** 1 Neurobiology Research Unit, Copenhagen University Hospital Rigshospitalet, Copenhagen, Denmark; 2 Faculty of Health and Medical Sciences, University of Copenhagen, Copenhagen, Denmark; 3 Department of Psychiatry and Psychotherapy, Medical University of Vienna, Vienna, Austria; 4 Department of Public Health, Section of Biostatistics, University of Copenhagen, Copenhagen, Denmark; 5 Psychiatric Centre Copenhagen, Copenhagen, Denmark; 6 Department of Drug Design and Pharmacology, University of Copenhagen, Copenhagen, Denmark

**Keywords:** Genetics of the nervous system, Neurotransmitters, Transporters in the nervous system

## Abstract

The serotonin transporter (5-HTT) critically shapes serotonin neurotransmission by regulating extracellular brain serotonin levels; it remains unclear to what extent 5-HTT levels in the human brain are genetically determined. Here we applied [^11^C]DASB positron emission tomography to image brain 5-HTT levels and evaluated associations with five common serotonin-related genetic variants that might indirectly regulate 5-HTT levels (*BDNF* rs6265, *SLC6A4* 5-HTTLPR, *HTR1A* rs6295, *HTR2A* rs7333412, and *MAOA* rs1137070) in 140 healthy volunteers. In addition, we explored whether these variants could predict in vivo 5-HTT levels using a five-fold cross-validation random forest framework. *MAOA* rs1137070 T-carriers showed significantly higher brain 5-HTT levels compared to C-homozygotes (2–11% across caudate, putamen, midbrain, thalamus, hippocampus, amygdala and neocortex). We did not observe significant associations for the *HTR1A* rs6295 and *HTR2A* rs7333412 genotypes. Our previously observed lower subcortical 5-HTT availability for rs6265 met-carriers remained in the presence of these additional variants. Despite this significant association, our prediction models showed that genotype moderately improved prediction of 5-HTT in caudate, but effects were not statistically significant after adjustment for multiple comparisons. Our observations provide additional evidence that serotonin-related genetic variants modulate adult human brain serotonin neurotransmission.

## Introduction

Serotonin neurotransmission mediates a multitude of brain functions, including neurodevelopment, behavior and cognition^1^. As such, identifying sources of individual variation in brain serotonin neurotransmission is relevant to identify mechanisms contributing to variation in behavior and possibly associated risks for disease. Dysregulation in brain serotonin signaling is implicated in a number of neuropsychiatric disorders such as anxiety and depression, which are known to have a prominent genetic component^2–5^. However, the contributions of genetic factors to individual differences in in vivo serotonin neurotransmission are not well understood.

The serotonin transporter (5-HTT) critically shapes serotonin neurotransmission as it facilitates serotonin reuptake, thereby regulating extracellular serotonin levels and associated receptor signaling^1^. It is also the pharmacological target for selective serotonin reuptake inhibitors (SSRIs), the most commonly prescribed class of antidepressants and anxiolytics^6^. 5-HTT availability can be visualized in humans in vivo using [^11^C]DASB positron emission tomography (PET)^7,8^.

Previous research has reported a link between 5-HTT levels and both healthy and pathological behavioral phenotypes. Increased 5-HTT availability (expressed in terms of binding potential, 5-HTT BP) has been linked to depressive symptoms severity in seasonal affective disorder^9^, greater negative affective bias^10,11^ and reduced amygdala reactivity to threat in healthy individuals^12,13^, whereas low 5-HTT availability has been associated with childhood abuse in patients with depression vs patients who did not experience childhood abuse^14^. Notably, some studies have speculated that 5-HTT availability may be used as a marker of serotonin tone and a histochemical marker for serotonergic projections^15,16^. Thus, identifying sources of variation in 5-HTT availability is relevant to advance our understanding of mechanisms driving individual variation in human behavior.

Evidence from PET studies supports a genetic effect on the brain distribution of receptors involved in serotonin function^17–19^. Similarly, genetic variation is likely to contribute to 5-HTT levels and activity. Multiple genetic variants have been hypothesized to affect brain serotonin signaling and behavior as well as brain function. Whereas human functional neuroimaging genetics with, e.g., BOLD fMRI, is challenging due to the inherently complex processes underlying brain function^20^, PET imaging provides a direct estimate of brain protein levels that is highly reproducible, making it a valuable target for relating to genetic variation.

5-HTTLPR, a common insertion/deletion polymorphism occurring in the promoter region of the 5-HTT gene (*SLC6A4*) is the most widely investigated 5-HTT variant. Nonetheless, although 5-HTTLPR was shown to affect 5-HTT gene expression in vitro in early studies^3^, PET studies in humans reported conflicting in vivo results^21–24^. In contrast, single nucleotide polymorphisms (SNP) in genes coding for proteins whose activity is related to that of 5-HTT, such as rs6265 (or Val66Met) in the neurotrophic factor (*BDNF*) gene^23^ and rs7333412 in the serotonin receptor 2A (5-HT_2A_) gene (*HT2AR)* were found to be associated with 5-HTT availability in healthy adults^25,26^. Specifically, the *BDNF* rs6265 met-carriers, who presumably have lower BDNF levels, showed increased 5-HTT availability in subcortical regions^23^, whereas the rs7333412 A-carriers showed reduced 5-HTT levels. BDNF is a common neurotrophin mediating neurodevelopmental, survival and plasticity functions whose activity levels can affect serotonin release ^27^ as well as 5-HTT expression in animal models^28^, while 5-HT_2A_ is a core regulator of excitatory serotonin neurotransmission. These findings suggest that genetic variants other than those in the *SLC6A4* gene may indirectly modulate 5-HTT availability by affecting serotonergic signaling via other pathways.In this framework, serotonin receptor 1A (5-HT_1A_, encoded by gene *HTR1A*), which is the principal inhibitory serotonin receptor that can inhibit serotonin release^29^, and monoamine oxidase A (MAOA, encoded by gene *MAOA*), the main enzyme involved in serotonin degradation^30^ can directly affect serotonin levels, which may in turn affect 5-HTT levels via its downregulation ^31^. Previous evidence reports that *HTR1A* rs6295 G-carriers have increased 5-HT_1A_ protein levels in the dorsal raphe, pointing to decreased serotonin tone that may in turn affect 5-HTT levels in humans ^34^. *MAOA* rs1137070 has been linked to increased MAOA mRNA levels and higher enzymatic activity, suggesting that this variant may directly affect serotonin levels^35^ and it was associated to SSRI treatment outcome^36,37^, suggesting a possible interaction with 5-HTT.

For explanatory purposes, an overview of the relationship between the serotonin-related genetic variants mentioned above and clinical or behavioral phenotypes is provided in Supplementary Table 1.

Previous studies have focused on the *association* between genetic variant and 5-HTT availability. However, although observing associations informs on group differences, it does not establish the ability to predict features at the individual level, which could be relevant for, e.g., personalized medicine strategies. To this end, exploring whether genetic variants assist in predicting 5-HTT availability can be of complementary value.

In this study we used the largest currently available dataset (N = 140) of [^11^C]DASB PET scans from the Cimbi database^38^ to explore the role of *BDNF* rs6265 and *SLC6A4* 5-HTTLPR(previously investigated in the same cohort^23^) rs7333412 in *HT2AR*^25,26^, rs6295 in *HTR1A*^32,39,40^, and rs1137070 in *MAOA*^36,41^ in 5-HTT availability in the healthy brain. First, we evaluated whether the genotypes examined were associated with 5-HTT availability. Next, we used random forest to determine whether genetic information predicted regional 5-HTT availability.

## Methods

### Participants

Cross-sectional data were included that was collected previously and were available from the Cimbi database^38^.

We selected healthy participants based on the following inclusion criteria: (1) availability of *BDNF* val66met (rs6265) and *SLC6A4* (5-HTTLPR and SNP rs23351) genotypes, (2) availability of blood samples for additional genotyping, (3) availability of a [^11^C]DASB PET scan before any intervention, 4) ⩽60 years of age (to avoid age-related biases in brain volumes, as partial volume effects become stronger after 60), 5) self-identification with European ancestry. In addition, we excluded participants who had the following: (1) diagnosis of a severe neurological or systemic disease; (2) diagnosis of a primary psychiatric disease; (3) substance or drug abuse, based on self-reported clinical history and neurological/physical examination.

We identified 140 healthy participants, 84 females and 56 males (mean age: 26.7 ± 7.2; range: 18–51). The sex imbalance is partly because some studies from which data are drawn included only males or females. Demographic data are described in Table 1.

**Table 1:** Demographic data of the 140 healthy volunteers included in the study and [^11^C]DASB scan information. Information regarding lifestyle was available only for subsets of the total participants: alcohol units per week (N = 131); Smoking status (N = 135); PSQI (N = 97); PSS (N = 126).

	**Total**	***BDNF* rs6265**		***SLC6A4* (5-HTTLPR, rs23351)**		***HTR1A* rs6295**		***HTR2A* rs7333412**		***MAOA* rs1137070**	
		**Val/val (G)**	**met- (A)**	**L**_**A**_**L**_**A**_	**S-**	**CC**	**G-**	**AA**	**G-**	**CC**	**T-**
**N**	140	90 (64.3%)	50 (35.7%)	41 (29.3%)	99 (70.7%)	33 (23.6%)	107 (76.4%)	87 (62.1%)	53 (37.9%)	75 (53.6%)	65 (46.4%)
**Age (mean ± s.d.)**	26.7 ± 7.2	26.4 ± 7.2	27.3 ± 7.1	26.4 ± 6.4	26.9 ± 7.5	25.7 ± 6.5	27.1 ± 7.4	27.3 ± 8.0	25.9 ± 5.5	25.7 ± 5.9	28.0 ± 8.3
**Sex (F/M)**	84/56	53/37	31/19	24/17	60/39	22/11	62/45	53/34	31/22	35/40	46/16
**PET scanner**											
**(A/H)**	42/98	30/60	12/38	13/28	29/70	7/26	35/72	23/64	19/34	26/49	16/49
**MRI scanner (T/V)**	81/59	52/38	29/21	22/19	59/40	16/17	65/42	52/35	29/24	48/27	33/32
**k2' (DASB kinetic modeling parameter)**	0.065 ± 0.013	0.065 ± 0.013	0.066 ± 0.012	0.066 ± 0.014	0.065 ± 0.012	0.066 ± 0.014	0.065 ± 0.013	0.065 ± 0.012	0.066 ± 0.014	0.065 ± 0.013	0.066 ± 0.013
**[**^11^**]DASB-injected mass (μg)**	3.2 ± 3.1	3.2 ± 2.9	3.2 ± 3.5	2.9 ± 2.9	3.3 ± 3.2	3.2 ± 3.2	3.2 ± 3.1	2.8 ± 2.7	3.8 ± 3.6	3.6 ± 3.5	2.7 ± 2.6
**[**^11^**]DASB-injected dose (MBq)**	552 ± 78	554 ± 72	548 ± 88	562 ± 73	548 ± 80	570 ± 53	546 ± 84	561 ± 65	537 ± 94	545 ± 82	560 ± 73
**cerebellum AUC (Bq ml**^**-1**^**)**	16,561 ± 4555	16,469 ± 4415	16,726 ± 4839	16,421 ± 4557	16,619 ± 4576	17,468 ± 3712	16,281 ± 4766	17,103 ± 4070	15,671 ± 5175	15,778 ± 4646	17,465 ± 4308
**Alcohol units per week**	7.8 ± 10.8 (N = 131)	8.2 ± 12.8	7.1 ± 5.9	10.4 ± 16.3	6.8 ± 7.5	6.2 ± 5.5	8.4 ± 12.0	7.5 ± 11.8	8.3 ± 9.1	9.3 ± 13.8	6.1 ± 4.9
**Smoking status (smokers/non smokers)**	39/96 (N = 135)	24/62	15/34	9/31	30/65	10/21	29/75	24/60	15/36	16/57	23/39
**PSQI**	3.9 ± 2.0 (N = 97)	3.8 ± 2.2	4.1 ± 1.7	3.8 ± 2.0	4.0 ± 2.0	3.8 ± 2.0	4.0 ± 2.0	4.1 ± 2.0	3.7 ± 2.0	3.5 ± 1.7	4.4 ± 2.1
**PSS**	9.3 ± 5.1 (N = 126)	9.3 ± 5.1	9.4 ± 5.0	8.4 ± 4.3	9.7 ± 5.3	8.4 ± 4.8	9.6 ± 5.1	9.8 ± 4.9	8.5 ± 5.3	8.8 ± 5.2	10.0 ± 4.9

Abbreviations: *BDNF*, brain-derived neurotrophic factor; *SLC6A4*, serotonin transporter gene; *5-HTTLPR*, serotonin-transporter-linked promoter region; *5-HT*_*1A*_*R*, serotonin receptor 1A; *5-HT*_*2A*_*R*, serotonin receptor 2A; *MAOA*, monoamine oxidase A; *F*, female; *M*, male; *PET*, positron emission tomography; *A*, GE-Advance PET scanner; *H*, HRRT PET scanner; *T*, Trio MRI scanner; *V*, Verio MRI scanner; *k2’* [^11^C]DASB kinetic modeling parameter; *μg*, microgram; *MBq*, megabecquerel; *Bq ml*^*-1*^, becquerel per milliliter; *AUC*, area under the curve (i.e., cerebellum reference region time activity curve); PSQI, Pittsburgh Sleep Quality Index; PSS, Cohen Perceived Stress Scale .

Subsets of the PET and genetic data included in the current study were collected as parts of multiple previous studies and have been included in previous publications. PET scans included in the current analyses were acquired between 2005 and 2015^11,18,42–46^. All research protocols were approved by the Ethics Committee of Copenhagen and Frederiksberg, Denmark ((KF) 01–124/04, (KF) 01–156/04, (KF) 01 2006–20, H-1–2010-085, H-1–2010-91, H-2–2010-108, amendments included). All participants provided written informed consent after receiving a detailed description of the respective study. All experimental procedures were carried out in compliance with the declaration of Helsinki.

Data included in the current study has been utilized in previous studies^9,10,23,45,47^, some of which focused on the relation between 5-HTTLPR and/or *BDNF* rs6265 [^11^C]DASB binding^23,45^.

#### Genotyping

No additional genotyping for the 5-HTTLPR (including rs25531) and *BDNF* rs6265 was performed beyond that which has been described previously^23,23,48,48,49^.

The three additional variants were determined from whole-blood derived genomic DNA using QIAamp DNA Blood Mini Kit (Qiagen, Valencia, CA). DNA concentration and purity levels were estimated using an UV–Vis spectrophotometer (Nanodrop 2000, Thermo Scientific).

The SNPs were determined using TaqMan SNP Genotyping Assay (Applied Biosystems, Foster City, CA) with genotype-specific probes (*BDNF* rs6265: C_11592758_10, *HTR1A* rs6295: C__11904666_10, *HTR2A* rs7333412: C__29235757, *MAOA* rs1137070: C___8878813_20). We performed real-time polymerase chain reaction for allelic discrimination using the LightCycler 480 Real-Time PCR System (Roche Diagnostics, IN).

#### MRI data acquisition

For each participant, high-resolution T1-weighted structural brain scans were acquired on either a Siemens Magnetom Trio 3 T (N = 81) or a Siemens Verio 3 T (N = 59) (Siemens, Erlangen, Germany magnetic resonance imaging (MRI) scanner. We used structural MRI scans for segmentation and to delineate regions of interest in the PET scans.

#### [^11^C]DASB PET data acquisition

We acquired PET scans for each participant on one of two PET scanners: 1) a Siemens ECAT high-resolution research tomography (HRRT) scanner operating in 3D-acquisition mode with an in-plane resolution of approximately 2 mm (N = 98) or 2) an 18-ring GE-Advance scanner (General Electric, Milwaukee, WI, USA) with a three-dimensional (3D) acquisition mode and an in-plane resolution of approximately 6 mm (N = 42). PET images were acquired on the HRRT scanner using a 6-min transmission scan followed by an intravenous bolus injection of [^11^C]DASB that was given over 20 s while a dynamic 90-min emission scan was acquired over 36 frames (6 × 10 s, 3 × 20 s, 6 × 30 s, 5 × 60 s, 5 × 120 s, 8 × 300 s, 3 × 600 s). Dynamic PET images were reconstructed with an iterative OP-OSEM3D method using resolution modeling (10 iterations, 16 subsets)^50,51^. Images acquired on the GE-Advance scanner involved a 10-min transmission scan followed by a bolus injection given over 12 s while a 90-min dynamic emission scan was acquired over 36 frames (same time frames as HRRT acquisitions). Dynamic PET images from the GE-Advance scanner were reconstructed using filtered back projection and were corrected for attenuation, dead-time and scatter with a 6-mm Hann filter.

For both scanners, we determined single-subject PET scan motion and realignment using an automatic image registration algorithm^52^. Next, we smoothed the PET images using a 10 mm (HRRT) or a 12 mm (GE-Advance) within-frame Gaussian filter and subsequently aligned the volumes. Using the scaled least squares cost function, we estimated the rigid translation/rotation parameters that align each PET frame to a reference frame with sufficient structural information (frame 26: 20-25 min post injection). We resliced non-filtered PET images and co-registered the high-resolution MR with PET using SPM (HRRT) or automatic image registration (GE-Advance). Co-registration was based on the mean of frames 10–26, i.e., a flow-weighted image; the accuracy of this step was confirmed visually. We automatically delineated brain regions on structural MRI scans using Pvelab^53^. We determined the time-activity curves across gray matter voxels within each region except for the midbrain region, where we used the mean time-activity across all voxels. We determined regional 5-HTT non-displaceable binding potential (BP_ND_) using kinetic modeling of regional time-activity curves in PMOD (Zurich, Switzerland) We applied the multilinear reference tissue model (MRTM/MRTM2) with a fixed k2’ estimated for each individual in a high binding region (caudate, putamen, and thalamus) and with cerebellum as reference region^54^. We calculated regional BP_ND_ values bilaterally, computing a volume-weighted mean from the right and left hemisphere.

#### Data analysis

We carried out the statistical analyses in R v4.1.2 (https://cran.r-project.org/). Consistent with previously related analyses, we considered regional 5-HTT BP_ND_ within caudate, putamen, midbrain, thalamus, hippocampus, amygdala and neocortex as our regions of interest^23^. We selected a single neocortical region (including orbitofrontal-, parietal- and occipital cortex and superior frontal-, pre/post central-, superior temporal- and middle/inferior frontal gyrus) because of previous evidence of high correlation between different cortical areas^55^. For all analyses, we grouped genotypes as follows: *SLC6A4* 5-HTTLPR and rs23351: L_A_L_A_ versus S’- carriers (individuals carrying at least one S- or one L_G_ allele); *HTR1A* rs6295: G-carriers (carrying at least one G-allele) vs CC homozygotes; *HTR2A* rs7333412: G- carriers vs AA homozygotes; *MAOA* rs1137070: T-carriers versus CC; *BDNF* rs6265: met-carriers versus val/val. In addition, we included age, sex, MRI and PET scanner type as covariates, consistent with previous findings^23^. We mean-centered all continuous variables. Although previous evidence showed a seasonal and body mass index (BMI) effect on 5-HTT availability^45,55^, no statistically significant effect of neither daylight minutes nor BMI on 5-HTT BP_ND_ was previously observed on the same cohort^23^ so these variables were not included as covariates. We considered additional lifestyle factors, including alcohol consumption (i.e., alcohol units per week), smoking status, total scores of the Pittsburgh Sleep Quality Index^56^ and of the Cohen Perceived Stress Scale^57^. None of these measures were significantly associated with regional 5-HTT BP_ND_ (*p* > 0.05) and were not included as covariates in the main analyses. For all the models, we set the statistical significance threshold to *p* < 0.05 (two-sided tests).

##### Association analyses

We fit a linear latent variable model (LVM) to evaluate associations between genotypes and 5-HTT BP_ND_ within our set of pre-defined brain regions (i.e., caudate, amygdala, hippocampus, putamen, thalamus, midbrain and neocortex) as described previously^23^. LVMs are a type of multivariate linear regression that effectively models associations in the presence of inter-correlated variables. In this case, 5-HTT BP_ND_ is highly intercorrelated between the brain regions that we considered^23^. Thus, using the *lava* package v 1.6.10^58^ in R, we modeled this shared correlation of regional 5-HTT BP_ND_ values with a latent variable (5-HTT_LV_). Next, we modeled all the genotype and covariate effects on 5-HTT_LV_. We included additional covariance links (caudate-putamen, amygdala-hippocampus and thalamus-midbrain) based on the model framework previously reported^23^. In addition, we used Wald tests of improvement in model fit to find additional paths. To control the false-discovery rate across all possible paths, we included the paths with a false-discovery rate of p_FDR_ < 0.05 calculated using the Benjamini–Hochberg test across all paths.

We used caudate as a reference region, so that the covariate effects reported here can be interpreted as effects on caudate BP_ND_ (corresponding to the reference scale).

**Figure 1:**
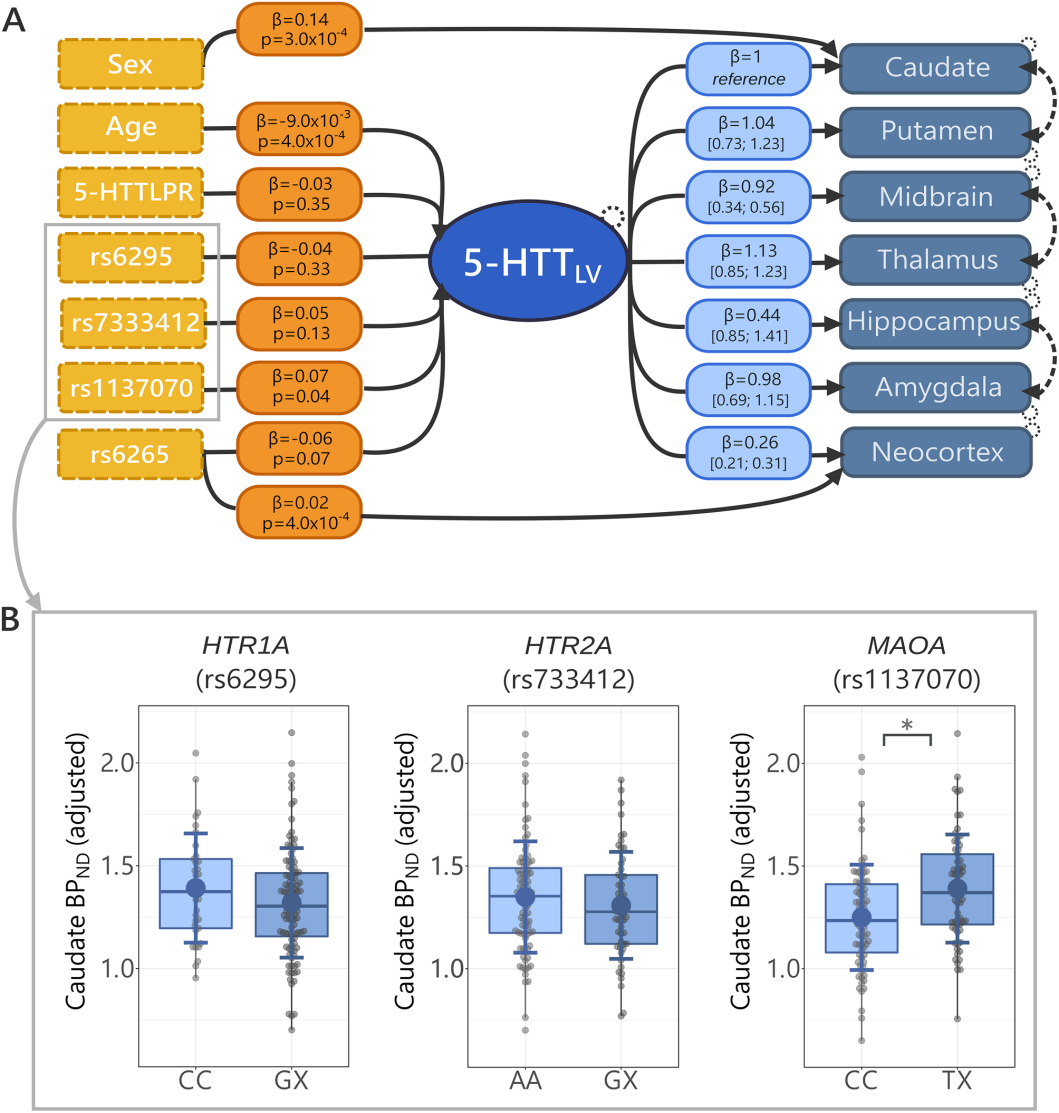
(**A**) Latent variable model (LVM) used to compute the association between each genotype independently from the other genotypes and 5-HTT BP_ND_ in caudate, putamen, midbrain, thalamus, hippocampus, amygdala and neocortex. PET and MRI scanner are not shown but were included as covariates. Yellow hatched boxes to the left represent the genetic variants and the covariates. The genetic variants depicted correspond to the following genes respectively: 5-HTTLPR- > *SLC6A4*, rs6295- > *HTR1A*, rs7333412- > *HTR2A*, rs1137070- > *MAOA*, rs6265- > *BDNF*. The orange boxes represent the covariate effects on the latent variable (5-HTT_LV_), which is represented by the central blue ellipse. Light blue boxes show the loadings on the latent variable of observed regional 5-HTT BP_ND_ (in the blue solid boxes to the right). β values in light blue and orange boxes indicate the parameter estimates for each model parameter with either its respective p-value (orange boxes) or 95% confidence interval (light blue). Hatched lines between regions indicate interregional shared correlations. Hatched circles on the brain regions represent the included error estimates. Arrows from the yellow to the blue boxes (sex- > caudate, rs6265- > neocortex) represent direct covariate effects on binding. All brain regions significantly loaded on to 5-HTT_LV_ (*p* < 1 × 10^–12^). (**B**) Boxplots showing representative effects of *HTR1A* rs6295, *HTR2A* rs7333412 and *MAOA* rs1137070 on caudate 5-HTT BP_ND_. The y axis represents 5-HTT BP_ND_, adjusted for age, sex, MRI scanner, and PET scanner. Gray dots represent 5-HTT BP_ND_ from each participant adjusted for age, sex, MRI and PET scanner. The larger solid dots and lines represent respective group means and ± 1 SD. The boxes represent datapoints from the 25% to the 75% quantile.5-HTT: serotonin transporter; *SLC6A4*: 5-HTT gene; *HTR1A*: serotonin receptor 1A gene; *HTR2A*: serotonin receptor 2 gene; *MAOA*: monoamine oxidase A gene; *BDNF*: brain-derived neurotrophic factor gene; MRI: magnetic resonance imaging (MRI); PET: positron emission tomography; BP_ND_: non-displaceable binding potential.

In addition, we estimated multiple linear regression models including all genotypes and covariates (age, sex, PET and MR scanner type) for each brain region and we used them to report percent differences in 5-HTT BP_ND_ between genotype groups. We reported all results with parameter estimates and 95% confidence intervals within brackets, including the associated units (Fig. 1).

Finally, we tested for a main effect of each variant using a likelihood ratio test comparing a model including all covariates and genotypes with a model including all covariates but not genotypes.

##### Prediction analyses

To determine whether genotype information predicts regional 5-HTT BP_ND_, we trained and tested a random forest model^59^. We used the *randomForest* package v.4.7–1.1 in R with default data sampling and model fitting parameters; this included p/3 sampled features per tree (where p represents the total number of features) and 500 trees per forest. As we were specifically interested in how well genetic information predicted 5-HTT BP_ND_, above and beyond other covariates, we constrained our feature set to include only the five genotypes and we evaluated the prediction of regional 5-HTT BP_ND_, adjusted for relevant covariates. First, we fitted a multiple linear regression model regressing each region, e.g., caudate 5-HTT BP_ND_, against age, sex, PET-scanner, and MR-scanner. The residuals from this multiple linear regression model were used as the outcome in our random forest machine learning models with genotype status for rs6265, 5-HTTLPR and rs23351, rs6295, rs7333412, and rs1137070 as model features. Prediction models were estimated using five-fold cross-validation; prediction of 5-HTT BP_ND_ on held out datasets (test data) within each fold was used to determine unbiased, predictive model performance. Model performance was calculated as the root mean-squared error (RMSE) of prediction on test data, across all folds. To account for fold-assignment related variance, model performance was assessed by repeating the five-fold cross-validation 10 times; overall performance was the mean of these 10 resamples. Statistical significance of performance was calculated from an empirical null distribution derived from 10,000 permutations of the resampled residuals. Notably, we performed five-fold cross-validation with 10 resamples within each of the 10,000 permutations so that each permutation more closely reflects our observed model structure. We fitted models for 5-HTT BP_ND_ in each of our seven regions of interest separately, i.e., seven different prediction models. In addition to expressing model performance in terms of RMSE, we express the percent change in RMSE of the prediction models accounting for genotype information (RMSE_genotype_) compared to the RMSE computed on residual 5-HTT BP_ND_ values (RMSE_residual_, i.e., ΔRMSE = 100*(RMSE_residual_ − RMSE_genotype_)/RMSE_residual_). Statistical significance (*p*-value) of model performance is expressed both uncorrected (p_unc_) and corrected (p_FWE_) for the seven models estimated, using Bonferroni-Holm, which controls the family-wise type-I error rate^60^.

## Results

### Genotyping

Genotype distribution and allelic frequencies are depicted in Table 1. rs6265, rs1137070, rs7333412, rs6295 and rs1137070 were in Hardy–Weinberg equilibrium (all *p* > 0.16). *MAOA* is x-linked so the allele frequency for rs1137070 in men was compared with the frequency in women using a chi-squared test to determine whether there were significant differences between allele frequencies in males and females. We did not find a statistically significant difference in allele frequencies between males and females (*P* = 0.67). Assessment of Hardy–Weinberg for the 5-HTTLPR is not valid because this polymorphism was an inclusion criterion for some studies from which these data are derived, i.e., participants were not sampled independent of 5-HTTLPR genotype^9,43^.

### Association analyses

The likelihood ratio showed that the LVM including genotype information was statistically significantly different from the LVM not including this information (*p* = 0.002), suggesting that genotype significantly contributed to the model.

The results from the LVM are depicted in Fig. 1. Consistent with previous observations, we observed that all regional 5-HTT BP_ND_ values loaded strongly on to 5-HTT_LV_ (all *p* < 10^–12^). After adding genetic variants and covariates (sex, age, PET and MRI scanner) to the model a Wald test did not support the inclusion of additional paths to the model (p_FDR_ > 0.05).

Within our final model, we found a statistically significant association between *MAOA* rs1137070 T-carriers (vs CC homozygotes) and 5-HTT_LV_ (estimate: 0.07, 95% CI: [8.08 × 10^–6^, 0.14], *p* = 0.039). Across regions, rs1137070 T-carriers showed ~ 2–11% higher 5-HTT BP_ND_ compared to CC individuals, the largest differences were observed in caudate (~ 11%) and putamen (~ 9%) and the lowest in amygdala (~ 2%).

As expected based on our previous evaluation of this model, we observed: (1) higher caudate 5-HTT BP_ND_ in males vs females (estimate: 0.14, 95% CI: [0.06, 0.21], *p* = 3.88 × 10^–4^); (2) a negative association between age and 5-HTT_LV_ (estimate: − 0.009, 95% CI: [− 0.015, − 0.0038], *p* < 0.001); (3) increased subcortical 5-HTT BP_ND_ in *BDNF* rs6265 met-carriers vs val/val individuals (estimate: − 0.06, 95% CI: [-0.04, -0.01], p = 0.07), corresponding to a 2–6% increase in 5-HTT BP_ND_ across subcortical areas. Conversely, an additional direct path from *BDNF* rs6265 to neocortex 5-HTT BP_ND_ effectively nullified the genetic effect on this brain region, which corresponds to the sum of indirect (*BDNF* rs6265—> 5-HTT_LV_—> neocortex BP_ND_) and direct (*BDNF* rs6265—> neocortex BP_ND_) effects. Although the estimate for statistical significance for rs6265 decreased in the presence of the three other genotypes, the effect size remained very similar, suggestive of independent main effects of *BDNF* rs6265 and *MAOA* rs1137070.

We did not observe evidence for an association between 5-HTTLPR (*p* = 0.35), *HTR1A* rs6295 (*p* = 0.33), nor *HTR2A* rs7333412 (*p* = 0.13) and 5-HTT_LV_.

### Prediction analyses

Across the seven regions evaluated, we observed that the set of genetic variant features slightly improved prediction of caudate 5-HTT BP_ND_ compared to the model not including genetic information (caudate: RMSE_residual_ = 0.262, RMSE_genotype_ = 0.266, ΔRMSE = 1.6%, p_unc_ = 0.036) (Table 2). However, this effect, and the effect in all other regions was not statistically significant after correction for seven models (Table 2).

**Table 2:** Uncorrected (p_unc_) and corrected (p_FWE_) p-values for each brain region; root mean squared error computed using residual 5-HTT BP_ND_ values (RMSE_residual_), using genotype information (RMSE_genotype_) and percent change in RMSE between RMSE_genotype_ and RMSE_residual_ (ΔRMSE). * indicates p_unc_ < 0.05.

Region	RMSE_residual_	RMSE_genotype_	ΔRMSE	p_unc_	p_FWE_
Caudate	0.266	0.263	1.61%	0.036*	0.252
Putamen	0.301	0.300	0.43%	0.112	0.560
Midbrain	0.251	0.253	− 1.01%	0.078	0.468
Thalamus	0.305	0.302	0.88%	0.205	0.652
Hippocampus	0.121	0.121	− 0.07%	0.468	0 .936
Amygdala	0.272	0.268	1.37%	0.163	0.652
Neocortex	0.056	0.057	− 1.75%	0.752	0.936

## Discussion

We observed that *MAOA* rs1137070 T-carriers had higher 5-HTT availability compared to CC individuals. T-carriers showed higher 5-HTT availability in all the seven brain regions examined, with the highest binding in caudate (~ 11%) and the lowest in amygdala (~ 2%). Conversely, variants in the *HTR1A* and *HTR2A* genes were not associated with 5-HTT availability. Despite observing evidence for a statistically significant association, the genetic variants were not significantly informative for predicting brain 5-HTT available above chance. Taken together, our findings support that genetic variation in the *MAOA* contributes to variation in brain 5-HTT availability in the healthy adult human brain.

MAOA degrades monoamines in the brain, including serotonin, for which it has a preferential affinity compared to its other substrates^61^. *MAOA* knock-out rodents have increased extracellular serotonin levels and abnormal affective behavior^30,62–64^ as well as reduced 5-HTT expression^63,65^, suggesting that genetically altered MAOA signaling can affect regulation of 5-HT levels, which may in turn modulate 5-HTT levels e.g. via downregulation^31^. Similarly, inhibition of MAO activity by monoamine oxidase inhibitors increases extracellular serotonin levels^62^ and is associated with reduced 5-HTT BP_ND_ in rhesus monkey and rats^66^. Notably, reductions in 5-HTT BP_ND_ following an acute pharmacologically-induced serotonin increase may not only reflect a downregulation of 5-HTT but also increased serotonin levels competing for the radioligand rather than a change in 5-HTT gene expression^67^.

The rs1137070 T-allele has been previously associated with lower MAOA enzymatic activity compared to the C-allele both in human fibroblasts in vitro and in post-mortem brains^35,68^. Studies on clinical populations have reported mixed findings^41,69,70^. Although some studies suggest a link between the T-allele and increased MAOA mRNA expression in peripheral blood of patients with depression compared to healthy controls^41^, as well as increased vulnerability to depression^41,69^, other studies reported an association between the C-allele and impaired antidepressant treatment outcome in women^70^.

Conversely, we found that healthy human rs1137070 T-carriers, previously associated with low MAOA activity compared to C-carriers, had greater 5-HTT availability. In this case, putatively lower MAOA activity would correspond to greater amounts of intra- and extracellular serotonin. We can speculate that increased 5-HTT availability reflects increased 5-HTT levels, which might be a compensatory mechanism put in place to reuptake the excess serotonin and maintain extracellular serotonin levels constant. Nonetheless, findings from preclinical research point towards an effect opposite to what we observed, whereas the studies in humans provided mixed findings. Thus, the ambiguity provided by previous evidence in humans does not allow us to draw conclusions about the relationship that we detected between *MAOA* rs1137070 and 5-HTT BP_ND_.

**Figure 2:**
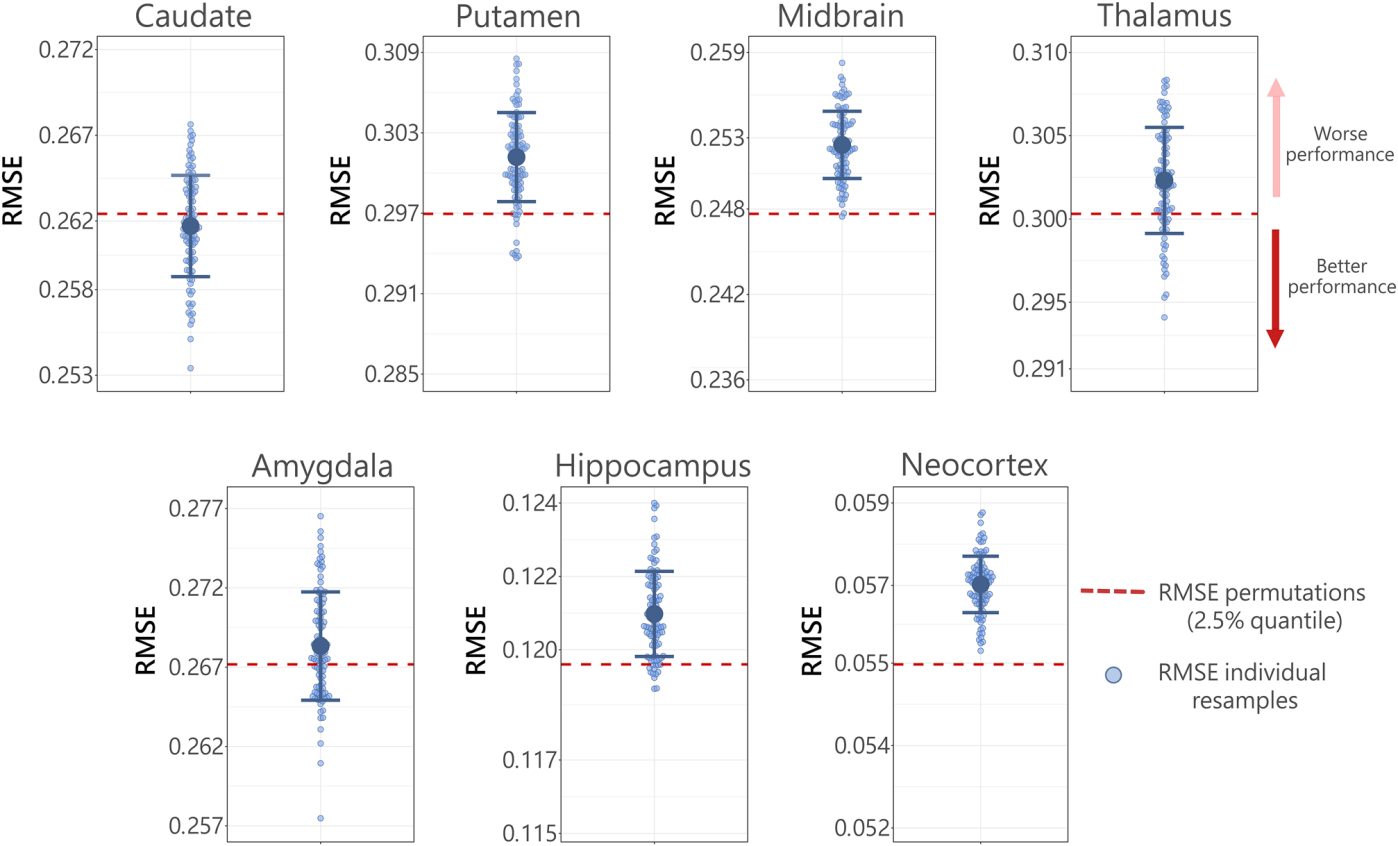
Random forest model performance. The light blue dots represent the individual RMSE values obtained from resampling (for display purposes, the distribution of the RMSE values from resampling is derived from a model run with 100 instead of 10 resamples) of the model including genotype information (RMSE_genotype_). The dark blue error plot displays the mean ± standard deviation of the distribution. The red hatched line indicates the 2.5% quantile of the average RMSE value derived from 10,000 permutations in the model that did not include genotype information (RMSE_residual_). Dark blue dots below the red hatched line indicate that the model performed significantly better than chance upon adding genotype information.

We did not observe evidence for an effect of *HTR2A* rs7333412 on 5-HTT availability. A previous study in patients with major depressive disorder, bipolar disorder, and healthy participants reported an effect on thalamus 5-HTT levels^25^. We did not replicate this effect in our study, as indicated by a comparison of Fig. 2 of their manuscript and our observed group differences. This is possibly because our study is based on a larger and more homogeneous cohort of healthy participants, whereas the previous study included patients with major depressive disorder and bipolar disorder as well as individuals with varying ethnic backgrounds. Taken together, our findings do not support that this *HTR2A* variant is associated with changes in 5-HTT availability in healthy adults.

Similarly, we did not observe evidence that *HTR1A* rs6295 is associated with 5-HTT availability, suggesting that whatever effects this polymorphism may have directly on the serotonin 1A receptor, those effects do not significantly modulate 5-HTT availability in healthy adults as measured with [^11^C]DASB PET.

Notably, we previously reported an association between *BDNF* rs6265 and 5-HTT BP_ND_, such that met-carriers showed reduced binding in subcortical areas compared to val-homozygotes^23^. Although this effect was marginally above our threshold for statistical significance in the current model, the effect size was very similar, suggesting independent contributions of *MAOA* rs1137070 and *BDNF* rs6265 to 5-HTT availability in healthy humans. As we reported previously, 5-HTTLPR was not significantly associated with 5-HTT BP_ND_ in this cohort^23,23^.

Regarding our prediction model, we observed that using genotype information led to a marginal improvement in predicting caudate 5-HTT BP_ND_ vs not using genotype information, but this effect was not significant after correcting for multiple comparisons. In addition, we could not predict 5-HTT BP_ND_ in any other brain regions. This limited performance may be because we evaluated only five variants, whereas genetically induced variation in 5-HTT levels likely stems from many variants.

Previous studies underscore the limited extent to which candidate variants exert main effects on complex behavioral traits or related features of brain activity^20,71^. Direct measures of discrete neurobiological features, e.g., serotonin transporter protein levels, may however be more susceptible to genetic variants that modulate the relevant neurotransmission pathways^72,73^. Nevertheless, alternative genetic analysis strategies such as GWAS would undoubtedly provide a more comprehensive evaluation of genetic contributions to 5-HTT levels in the human brain. However, an exploratory GWAS requires either thousands of datasets or very large effect sizes (i.e., > 20% difference in 5-HTT BP_ND_, Cohen’s d > 1) to establish statistical significance. GWAS-based polygenic risk scores, e.g., for psychiatric disorders or independent-dataset, hypothesis-generation via, e.g., expression quantitative trait loci (eQTL) databases may instead provide informative and statistically viable strategies for resolving genetic contributions to variation in brain serotonin neurotransmission measured with PET. Our cohort of 140 healthy participants stands as the largest single database of 5-HTT PET brain scans in the world. Future studies probing genetic contributions to brain serotonin-related PET scans would likely benefit from pooling data via, e.g., OpenNeuro (https://openneuropet.github.io/).

Although genetic variation is plausible and partially supported by our findings, environmental factors are also likely to contribute to 5-HTT levels^67^. A previous study based on the same cohort used for the present study reported no association between 5-HTT levels and daylight minutes or body mass index, contrarily to what reported by earlier studies based on smaller cohorts, part of which are included in our analyses^42,55^. In addition, we could not find any effect of variables reflecting lifestyle measures such as smoking, alcohol consumption, sleep and perceived stress on 5-HTT levels, suggesting that such environmental contributions in this cohort did not confound the observed genetic effects.

All participants self-identified with European ancestry. Self-reports of ancestry can be inaccurate^74^ and the lack of ethnic diversity in our sample limits the generalizability of our findings.

Notably, we exclude that the association between 5-HTT availability and the rs6265 and rs1137070 genotypes is due to a direct effect of genotype on BP_ND_. BP_ND_ is proportional to the amount of target proteins available for binding (B_avail_), i.e. 5-HTT, the affinity constant of the radioligand for its target (K_D_) and the free fraction of ligand in the non-displaceable tissue compartment (f_ND_)^75^. We infer the observed genetic effects to be primarily related to change in B_avail_, although we cannot rule out effects on K_D_ or f_ND_. Nonetheless, this seems unlikely because rs6265 and rs1137070 are proximal to *SLC6A4*, but a two scan study structure could more directly disentangle effects of B_avail_ and K_D_^76^.In conclusion, we report evidence for the association between *MAOA* rs1137070 genotype and brain 5-HTT availability. We did not observe evidence for an effect of *HTR1A* and *HTR2A* variants previously associated with brain serotonin markers, suggesting that their contribution may not be relevant to 5-HTT availability in the healthy adult human brain. Future studies considering additional genetic variants as well as environmental factors in larger datasets are critical for improving our understanding of the factors shaping serotonergic neurotransmission in health and disease.

### Supplementary Information

Supplementary Table 1.

## Data Availability

The R code employed for statistical analyses can be made available upon request to the corresponding author (patrick.fisher@nru.dk). Data can be made available upon reasonable request via this form (https://cimbi.dk/index.php/documents/category/3-cimbi-database) and with an appropriate data sharing agreement.

## Code availability

The R codes used for the latent variable model and the random forest model described in this manuscript can be made available upon request.
